# The Pharmaceutical Society and the War

**Published:** 1915-05-29

**Authors:** 


President.

26 St. James's Place, S.W.
May 25, 1915.

### The Pharmaceutical Society and the War.

THE annual meeting of the members of the Pharmaceutical Society of Great Britain was held on May 19, 1915, at 17 Bloomsbury Square, London. Mr. Edmund White, B.Sc., F.I.C. (President of the Society), was in the chair. The seventy-fourth annual report of the Council was submitted, and gave evidence of the vast amount of work carried out by this body on behalf of the some 8,000 pharmacists whom it represents. Mention was made of the effect of the war on the Society's School of Pharmacy and that the Council have arranged to keep open the places of demonstrators, etc., who have joined his Majesty's forces.

The President, in an able *résumé* of the activities of the Society, referred to the scheme for the supply of drugs, etc., to necessitous dependants of soldiers and sailors, which was carried out in conjunction with the medical profession. The Pharmaceutical Society had agreed to bear all the expense of checking the prices of prescriptions; individual chemists on their part agreed to dispense all medicines without charging any fee. As with many such schemes, there has been some abuse, a small percentage of dependants who are not really necessitous obtaining treatment. The Society had also joined with the British Medical Association in a scheme for rendering aid to Belgian doctors and pharmacists. Pharmacists' subscriptions have already amounted to £1,750.

The President, speaking on National Insurance, referred to the action taken by the Government in granting a sum to reduce the chemists' heavy losses by the discounting of their bills, and said that whilst every pharmacist would not, even now, receive payment for his work, yet it would be evident that the action taken by the Society should reduce the item of loss considerably.

### MEDICAL ALCOHOL.

Of particular interest was the President's statement of the action taken with regard to the proposed new spirit duties, in the endeavour to induce the Government to exclude medicinal alcohol from the provisions of the Bill. In conjunction with Dr. Cox, of the B.M.A., action had been taken, and finally the Chancellor accepted an amendment to allow "doctors, hospitals, chemists, and corporate bodies entitled to carry on the business of chemist and druggist" being exempted from the duty on immature spirits. Mr. White referred in high terms of praise to the invaluable service rendered by Mr. W. S. Glyn-Jones, M.P. (Parliamentary Secretary), saying that the results achieved were, in a large measure, due to his persistent intervention in the debate on the subject in the House of Commons.

Reference to the status of pharmacists in the Army was made, but it was hardly expected to get any definite result at the present time.

As is usual at annual meetings of the Society, many members criticised adversely some of the actions of their Council, but the fact that this year there is no contest for election to the Council would seem to indicate that the general body of members are satisfied.

